# Changes in Plant Species Richness Induce Functional Shifts in Soil Nematode Communities in Experimental Grassland

**DOI:** 10.1371/journal.pone.0024087

**Published:** 2011-09-01

**Authors:** Nico Eisenhauer, Varvara D. Migunova, Michael Ackermann, Liliane Ruess, Stefan Scheu

**Affiliations:** 1 Department of Forest Resources, University of Minnesota, St. Paul, Minnesota, United States of America; 2 K.I. Skryabin All–Russian Institute of Helminthology, Moscow, Russia; 3 Humboldt University Berlin, Institute of Biology, Berlin, Germany; 4 Georg August University Göttingen, J.F. Blumenbach Institute of Zoology and Anthropology, Göttingen, Germany; Freie Universität Berlin, Germany

## Abstract

**Background:**

Changes in plant diversity may induce distinct changes in soil food web structure and accompanying soil feedbacks to plants. However, knowledge of the long-term consequences of plant community simplification for soil animal food webs and functioning is scarce. Nematodes, the most abundant and diverse soil Metazoa, represent the complexity of soil food webs as they comprise all major trophic groups and allow calculation of a number of functional indices.

**Methodology/Principal Findings:**

We studied the functional composition of nematode communities three and five years after establishment of a grassland plant diversity experiment (Jena Experiment). In response to plant community simplification common nematode species disappeared and pronounced functional shifts in community structure occurred. The relevance of the fungal energy channel was higher in spring 2007 than in autumn 2005, particularly in species-rich plant assemblages. This resulted in a significant positive relationship between plant species richness and the ratio of fungal-to-bacterial feeders. Moreover, the density of predators increased significantly with plant diversity after five years, pointing to increased soil food web complexity in species-rich plant assemblages. Remarkably, in complex plant communities the nematode community shifted in favour of microbivores and predators, thereby reducing the relative abundance of plant feeders after five years.

**Conclusions/Significance:**

The results suggest that species-poor plant assemblages may suffer from nematode communities detrimental to plants, whereas species-rich plant assemblages support a higher proportion of microbivorous nematodes stimulating nutrient cycling and hence plant performance; i.e. effects of nematodes on plants may switch from negative to positive. Overall, food web complexity is likely to decrease in response to plant community simplification and results of this study suggest that this results mainly from the loss of common species which likely alter plant – nematode interactions.

## Introduction

Biodiversity is declining at an unprecedented speed [1], [2] resulting in significant changes in the functioning of ecosystems [3]. Plant diversity experiments in temperate grasslands suggest that aboveground ecosystem functions increase with plant diversity [4]–[6]. Although they received much less attention, there is increasing evidence that changes in the diversity within the producer level propagate into consumer levels above and below the ground, altering their performance, diversity and functioning [7]–[10]. Such cascading effects may have strong feedbacks on primary producers as well as on ecosystem processes [7], [11]. In the present paper we investigated the functional composition of soil nematode communities – a model group for soil animal food webs [12]–[15] – in an experimental plant diversity gradient in order to explore if changes in plant diversity and functional composition lead to functional shifts in soil food webs.

There is increasing evidence that microbial communities and ecosystem services of soils are beneficially affected by plant diversity [8], [16]–[18]. However, the role of plant diversity for soil food web structure is largely unknown (but see [10]). Importantly, as soil food webs respond in a decelerated way to changes in plant community composition [8], [9], [19], [20], we lack a comprehensive understanding of functional shifts below the ground.

Analyses of microbial communities in plant diversity experiments indicate that particularly fungal biomass increases with plant diversity [10], [17], [18], pointing to increasing importance of the fungal energy channel in species-rich plant assemblages. There are strong linkages between nematodes and their bacterial or fungal food resources and the nature and abundance of these resources can be monitored by faunal analysis of nematode communities, e.g. the fungal-to-bacterial feeder ratio or the channel index [21]. However, results from field studies on nematodes do not support an enhanced fungal energy pathway with increased plant diversity [22,23; but see 24]. Rather, they highlight the relevance of the presence of certain plant species and/or functional groups for the functional composition of nematode communities [22], [23], [25], [26]. In addition to the ratio between fungal and bacterial feeders, belowground functional shifts may be indicated by the ratio between predators and plant feeders. Haddad et al. [7] reported that loss of plant species shifted a predator-dominated aboveground food web to being herbivore-dominated. The low density and diversity of predators in species-poor plant communities likely indicates a decrease in food web complexity.

Moreover, Haddad et al. [7] found a close positive relationship between plant species richness and aboveground consumer diversity which was primarily due to the loss of rare species in low diverse plant assemblages. The authors suggested that high plant diversity ensures habitat and food resources for rare species and that rare herbivore species may have been associated with rare plant species in polycultures. Similarly, particularly rare plant species have been reported to get lost after N fertilization [27], [28]. If the positive relationship between plant diversity and soil animal diversity, observed in recent studies [9], [10], is due to the loss of rare species has not been investigated. The loss of rare and thus functionally inconspicuous species may cause the often reported saturating relationship between species richness and ecosystem functioning according to the redundancy hypothesis [29].

We studied nematode communities in a grassland plant diversity gradient three and five years after establishment of experimental plots to investigate if (1) plant diversity is related to belowground diversity, particularly due to the lack of rare taxa in species-poor plant communities, and (2) changes in plant diversity induce alterations in the functional composition of soil food webs, e.g. an increasing relevance of fungal feeding nematodes and predators.

## Materials and Methods

### Experimental setup

**Figure 1:**
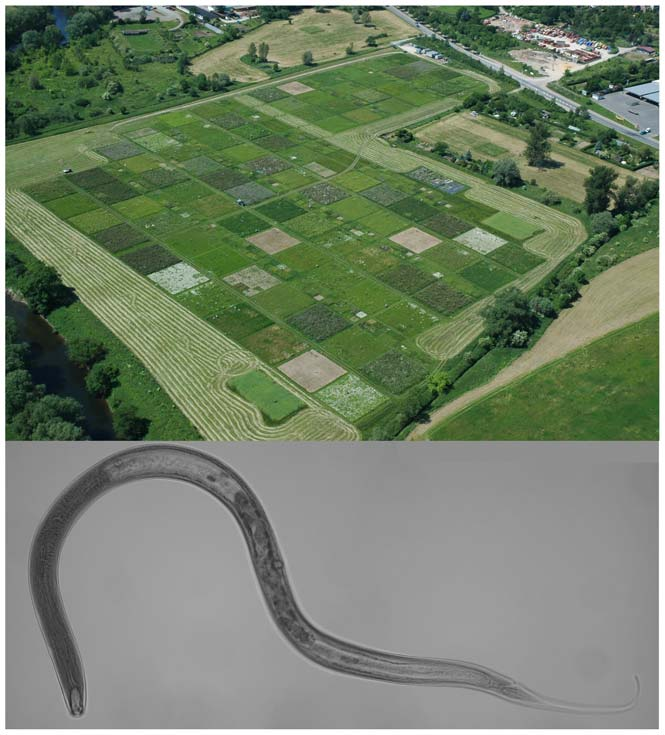
Photograph of the field site of the Jena Experiment and an exemplary nematode. The upper photograph was taken in 2006 showing the main experimental plots (20×20 m) varying in plant species richness (1, 2, 4, 8, 16, and 60 species) and plant functional group richness (1, 2, 3, and 4 functional groups). The field site is located on the floodplain of the Saale river at the northern edge of Jena (Thuringia, Germany). Photo by Christoph Scherber, Winfried Voigt, Alexandra Weigelt & the Jena Experiment. The lower photograph shows the nematode *Mononchus* sp., a predator species found at the field site of the Jena Experiment. Photo by René Seiml-Buchklinger.

The study was conducted in the frame of the Jena Experiment, a large field experiment exploring the role of biodiversity for element cycling and trophic interactions in grassland communities (Fig. 1; [30]). The study site is located on the floodplain of the Saale river at the northern edge of Jena (Thuringia, Germany). Mean annual air temperature is 9.3°C and annual precipitation is 587 mm [31]. The site had been used as an arable field for 40 years; the soil is characterized as Eutric Fluvisol [32]. The experiment was established in May 2002 to represent Central European mesophilic grassland of different diversity traditionally used as hay meadow (Arrhenatherion community). A pool of 60 native plant species was used to establish a gradient of plant species richness (1, 2, 4, 8, 16 and 60 species) and plant functional groups richness (1, 2, 3 and 4 functional groups) in a total of 82 plots of 20×20 m [30]. Using above- and belowground morphological traits, phenological traits and the ability for N_2_ fixation, plant species were aggregated into four plant functional groups: grasses (16 species), small herbs (12 species), tall herbs (20 species) and legumes (12 species; [30]). Experimental plots were mown twice a year (June and September), as is typical for hay meadows in Central Europe, and weeded twice a year (April and July) to maintain the target species composition. Plots were assembled into four blocks following a gradient in soil characteristics perpendicular to the Saale river. Each block contained an equal number of plots of all plant species and plant functional group richness levels. Further information on the design and setup of the Jena Experiment is given in Roscher et al. [30].

### Nematode sampling, extraction and identification

Soil samplings were performed in autumn (October) 2005 and spring (May) 2007, i.e. three and five years after the establishment of the experimental plots. In 2005 the sampling was performed on plots of the whole plant species richness gradient (n = 73; Table 1, Table S1) and nematodes (Fig. 1) were identified to genus level. Of the 82 plots of the Jena experiment we used 73 with well established plant communities excluding e.g., some monocultures with low coverage of the target species. In 2007 only experimental plots containing 1 (8 plots), 4 (7 plots) and 16 plant species (7 plots) in blocks 1 and 2 were sampled (n = 22; Table 1), and nematodes were assigned to the trophic groups plant feeders, bacterial feeders, fungal feeders, predators and omnivores according to Yeates et al. [33] and Bongers and Bongers [34]. At each sampling campaign, five soil samples were taken per plot (diameter 2 cm, depth 5 cm), pooled and stored at 5°C for <1 week. From 10 g soil fresh weight of these samples nematodes were extracted using a modified Baermann method [35]. After an extraction time of 30 h, nematodes were preserved in 4% formaldehyde. The number of extracted nematodes in each sample was counted and 10% of the individuals (but not less than 100 individuals, if available) were assigned to trophic groups (2005 and 2007) and identified to genus level (2005). No specific permits were required for the described field study. Our study did not involve endangered or protected species.

**Table 1:** Design of the Jena Experiment.

		Plant species richness						
		1	2	4	8	16	60	Plots
Plant functional group richness	1	*15* **(8)**	*4*	*4* **(2)**	*4*	*2* **(1)**	*-*	*29* **(11)**
	2	*-*	*8*	*3* **(2)**	*3*	*4* **(2)**	*-*	*18* **(4)**
	3	*-*	*-*	*4* **(2)**	*4*	*3* **(2)**	*-*	*11* **(4)**
	4	*-*	*-*	*4* **(1)**	*4*	*4* **(2)**	*3*	*15* **(3)**
Plots		*15* **(8)**	*12*	*15* **(7)**	*15*	*13* **(7)**	*3*	*73* **(22)**

Combinations of plant species and plant functional group richness, and the number of plots per diversity level at the Jena Experiment field site (see Table S2 for more details). Plots sampled in autumn 2005 are given in italics and plots sampled in spring 2007 are given in bold (brackets). For more details on the design of the Jena Experiment see Roscher et al. (2004).

### Calculations and indices

For a detailed investigation of plant community effects on nematode diversity we determined total number of taxa and the Shannon-Wiener index as well as number of nematode taxa of rare, intermediate and common species, and of the colonizer-persister groups *cp*-1 to *cp*-5 (all 2005). Taxa occurring on maximal 14 plots (<20%; 17 taxa) were grouped as “rare”, those occurring on 15 to 30 (21–41%; 17 taxa) as “intermediate”, and those occurring on at least 31 plots (>42%; 17 taxa) were regarded as “common” (see Table S2). This proxy measure for “rareness” correlated well with the density of the taxa (R^2^ = 0.71; P<0.001), suggesting that common species were also more dominant than rare species. This grouping was chosen to represent each group (rare, intermediate and common) with an equal number of species. Moreover, we used the colonizer-persister grouping of Bongers and Bongers [34] and Ferris et al. [12]: *cp*-1 (4 taxa), *cp*-2 (20), *cp*-3 (8), *cp*-4 (8) and *cp*-5 (4). The colonizer-persister grouping pools nematode taxa with similar response to changes in their environment, i.e. life-history characteristics and feeding behaviour. The scale ranges from enrichment opportunists (*cp*-1), i.e. *r*-strategists in a looser sense to taxa sensitive to disturbance (*cp*-5), i.e. *K*-strategists *sensu lato* [34], [36], [37].

In order to indentify plant community effects on the functional composition of nematode communities we calculated the ratio between fungal (FF) and bacterial feeders (BF) as FF/(FF+BF), the ratio between microbivore nematodes (FF and BF) and plant feeders, and the ratio between predators and plant feeders in 2005 and 2007. While the ratio between fungal and bacterial feeders provides information on changes in the relevance of energy channels, the ratio between microbivores and plant feeders indicate the net effect of nematode communities on plant productivity [38], [39]: a ratio >1.0 suggests positive net effects of nematodes outweigh the negative ones. The ratio between predators and plant feeders represents a proxy measure of food web complexity and the ability of communities to control herbivore populations that can become pests [7]. Moreover, we calculated the maturity index, channel index, enrichment index and structure index in 2005, representing common indices using weighed abundances of nematodes according to their trophic affiliation in order to explore functional changes in food web structure [12], [21]. Overall effects of plant species richness on nematode density and diversity in 2005 have been published elsewhere [10]. Further, it should be noted that plant community effects on nematode densities reported in Eisenhauer et al. [40] cannot be compared with those reported here, since the former study comprised data from untreated subplots and subplots with nematicide application. In the present paper we focus in more detail on plant community effects on functional changes in food web structure and on the variability of community functioning between the two sampling dates in autumn 2005 and spring 2007.

### Statistical analyses

Data on nematode density were log-transformed to meet the requirements of General Linear Model (GLM; normality and homoscedasticity of errors). GLM (type I sum of squares) was used to test the effects of block (BL), plant species richness (SR), plant functional group richness (FR), and presence/absence of grasses (GR), small herbs (SH), tall herbs (TH) and legumes (LE) on the density (total, plant feeders, bacterial feeders, fungal feeders, predators and omnivores; 2005 and 2007) and diversity of nematodes (taxa richness of total nematodes, rare, intermediate, common, *cp*-1, *cp*-2, *cp*-3, *cp*-4, *cp*-5 and Shannon-Wiener index; 2005), the maturity index (2005), enrichment index (2005), structure index (2005), on the ratio between fungal and bacterial feeders (2005 and 2007), on that between microbivores and plant feeders (2005 and 2007), and on that between predators and herbivores (2005 and 2007). Data had to be analyzed separately for 2005 (n = 73) and 2007 (n = 22) due to differences in sample size. *F*-values given in text and tables refer to those where the respective factor was fitted first [41]. BL was always fitted first, followed by plant community properties. If not explained otherwise, SR and FR were tested as linear factors.

In order to compare the ratios between fungal and bacterial feeders as well as between microbivores and plant feeders in 2005 and 2007, we used Wilcoxon Matched Pairs Test (non-parametric test for the comparison of dependent variables). To explore if the ratio between microbivores and plant feeders differs from neutral effects ( =  1.0), we performed one sample *t*-tests. To compare the two sampling dates we only used plots which were sampled in both years. We did not correct for multiple statistical tests considering the mathematical and logical argumentation by Moran [42]. All statistical analyses were performed using STATISTICA 7 (Statsoft).

## Results

Generally, soil heterogeneity (block) significantly impacted several nematode taxa and had to be considered in the statistical analyses. However, the effects are not presented and discussed in detail as this study focuses on plant community impacts on nematodes rather than on the role of abiotic factors. A list of nematode taxa, trophic group affiliation and occurrence on the experimental field site is given in Table S2.

### Nematode density

**Figure 2:**
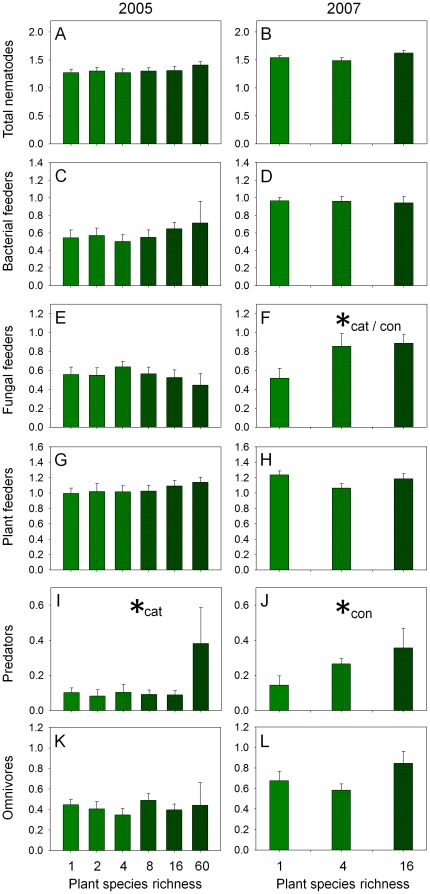
Plant diversity effects on nematode trophic groups. Effect of plant species richness on the density of (A, B) total nematodes, (C, D) bacterial feeders, (E, F) fungal feeders, (G, H) plant feeders, (I, J) predators, and (K, L) omnivores [log10 (number g^−1^ soil dry weight)] in autumn 2005 (A, C, E, G, I, K) and spring 2007 (B, D, F, H, J, L). Means ± standard error; *  =  significant (*P*≤0.05), cat  =  categorical factor, con  =  continuous factor. Darker shading of green indicates increasing plant species richness.

Total nematode density was not affected significantly both in 2005 (21±12 individuals g^−1^ soil dry weight) and 2007 (36±11 individuals g^−1^ soil dry weight) by plant community properties (Table 2, Fig. 2A, B). In 2005 neither plant species nor plant functional group richness significantly affected nematode population density or the density of individual trophic groups (Table 2; Fig. 2A, C, E, G, K), despite a significant effect of plant species richness on the density of predators (categorial factor: *F*_5,64_ = 2.64, *P* = 0.031; Fig. 2I). From the different plant functional groups only the presence of grasses affected the density of bacterial feeders negatively (−37%; Table 2). In 2007 the density of fungal feeders (linear factor: Table 2; categorial factor: *F*_2,17_ = 4.09, *P* = 0.035; Fig. 2F) and predators (Fig. 2J) increased significantly with plant species richness, whereas the other nematode trophic groups did not respond significantly to plant community properties (Table 2; Fig. 2B, D, H, L).

**Table 2:** Plant community effects on nematodes.

		BL	SR	FR	GR	SH	TH	LE
**2005**								
Nematode density		2.76	0.57	0.48	0.44	0.00	1.10	0.00
	Plant feeders	3.13*	0.96	0.20	0.22	0.01	0.31	0.71
	Bacterial feeders	3.15*	0.86	0.18	**6.92** * ↓	0.09	0.21	2.92
	Fungal feeders	0.20	0.28	0.19	0.42	0.11	0.28	0.21
	Predators	0.36	2.25	0.28	2.10	0.70	0.01	0.27
	Omnivores	6.60**	0.04	0.15	0.00	0.35	2.37	0.00
Nematode richness		5.09**	**4.84** * ↑	0.13	0.15	0.79	0.33	1.00
	Rare	5.78**	0.05	0.59	0.01	**4.24** * ↓	0.05	0.45
	Intermediate	2.17	0.29	0.51	0.03	0.42	0.55	0.21
	Common	3.00*	**7.47** ** ↑	0.18	0.00	0.03	0.08	0.98
	*cp*-1	1.47	0.51	0.09	0.62	0.28	0.22	0.02
	*cp*-2	12.12***	0.49	0.34	1.35	0.34	0.08	0.40
	*cp*-3	2.75	**6.08** * ↑	0.21	1.45	0.02	0.00	0.01
	*cp*-4	1.70	0.01	1.53	0.19	**5.26** * ↓	0.55	0.08
	*cp*-5	7.48***	1.89	0.00	0.14	0.34	2.31	0.44
Shannon-Wiener index		2.69*	**3.67** * ↑	0.00	0.06	0.00	0.99	1.77
Maturity index		4.16**	0.19	0.01	1.53	0.50	2.48	0.27
Channel index		2.67	1.06	0.00	0.79	0.11	0.25	0.40
Enrichment index		0.71	0.26	0.06	0.02	1.18	1.09	0.54
Structure index		3.04*	0.21	0.05	2.93	0.00	0.18	0.01
FF/(FF+BF)		2.39	1.37	0.00	2.34	0.47	0.03	0.84
(FF+BF)/PF		1.90	0.41	0.05	3.12	1.13	0.10	1.20
PR/PF		2.78*	0.00	2.02	0.12	0.03	0.74	3.77
**2007**								
Nematode density		0.03	1.36	0.32	2.16	0.19	1.61	1.12
	Plant feeders	0.60	0.30	0.01	0.34	0.05	0.04	0.10
	Bacterial feeders	0.00	0.07	0.00	0.10	0.27	0.44	0.50
	Fungal feeders	1.61	**7.87** * ↑	1.67	0.78	3.25	2.31	1.62
	Predators	0.00	**4.52** * ↑	0.15	0.22	0.14	0.09	0.08
	Omnivores	4.11	1.90	0.12	0.91	0.47	0.15	0.49
FF/(BF+FF)		2.98	**11.63** ** ↑	2.25	0.79	2.82	2.64	0.63
(FF+BF)/PF		0.13	1.35	0.01	0.13	0.00	0.28	0.38
PR/PF		2.33	**4.63** * ↑	0.26	0.74	0.10	0.01	0.01

*F*-values of GLMs for the effect of block (BL), plant species richness (SR), plant functional group richness (FG), presence/absence of grasses (GR), small herbs (SH), tall herbs (TH) and legumes on total nematode density and on the density of different trophic groups of nematodes (autumn 2005 and spring 2007), the ratio between fungal feeders (FF) and bacterial feeders (BF; autumn 2005 and spring 2007), the ratio between FF+BF and plant feeders (PF), the ratio between predators (PR) and plant feeders, nematode taxon richness, nematode taxon richness of rare, intermediate and common taxa, and of taxa belonging to different colonizer-persister groups [12], [34], Shannon-Wiener index, maturity index, channel index enrichment index and structure index (all in autumn 2005).

Significant effects (*P*≤0.05) are given in bold. n_2005_ = 73, n_2007_ = 22, degrees of freedom: BL = 3 (2005) or 1 (2007), SR, FR, GR, SH, TH, LE = 1;

↑  =  increase with increasing plant species richness,

↓  =  decrease in the presence of a certain plant functional group,

***  =  *P*≤0.001,

**  =  *P*≤0.01,

*  =  *P*≤0.05.

### Nematode diversity

**Figure 3:**
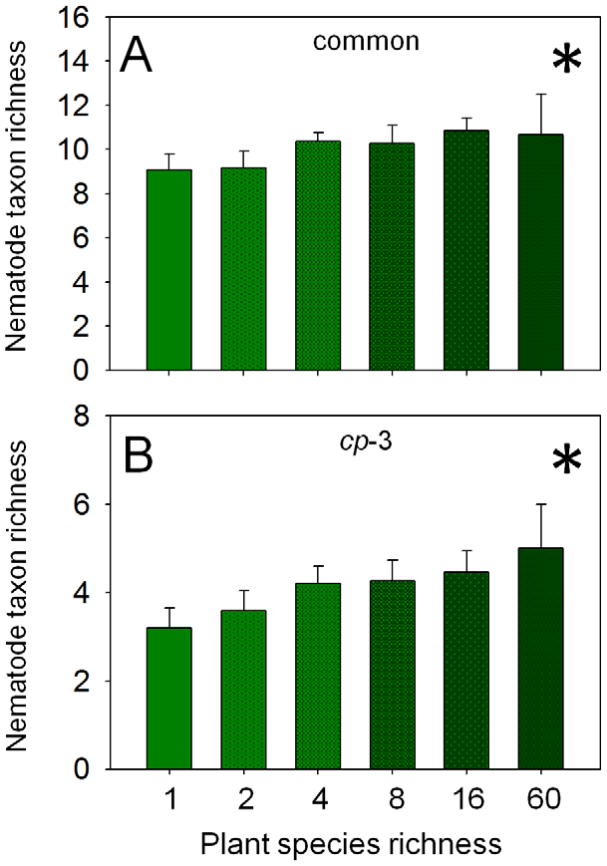
Plant diversity effects on nematode taxon richness. Effect of plant species richness on the taxon richness of (A) common nematode taxa (see text for details) and (B) taxa belonging to the colonizer-persister group 3 (see text for details) in autumn 2005. Means ± standard error; *  =  significant (*P*≤0.05). Darker shading of green indicates increasing plant species richness.

Total number of nematode taxa and the Shannon-Wiener index increased significantly with plant species richness (Table 2). More detailed analyses indicated that the increase in nematode diversity occurred in common taxa and taxa belonging to the *cp*-3 group (Table 2, Fig. 3A, B). The diversity of rare taxa (−14%) and taxa belonging to the *cp*-4 group (−17%) decreased significantly in the presence of small herbs, whereas the other nematode groups did not respond significantly to any plant community properties (Table 2).

### Functional indices

**Figure 4:**
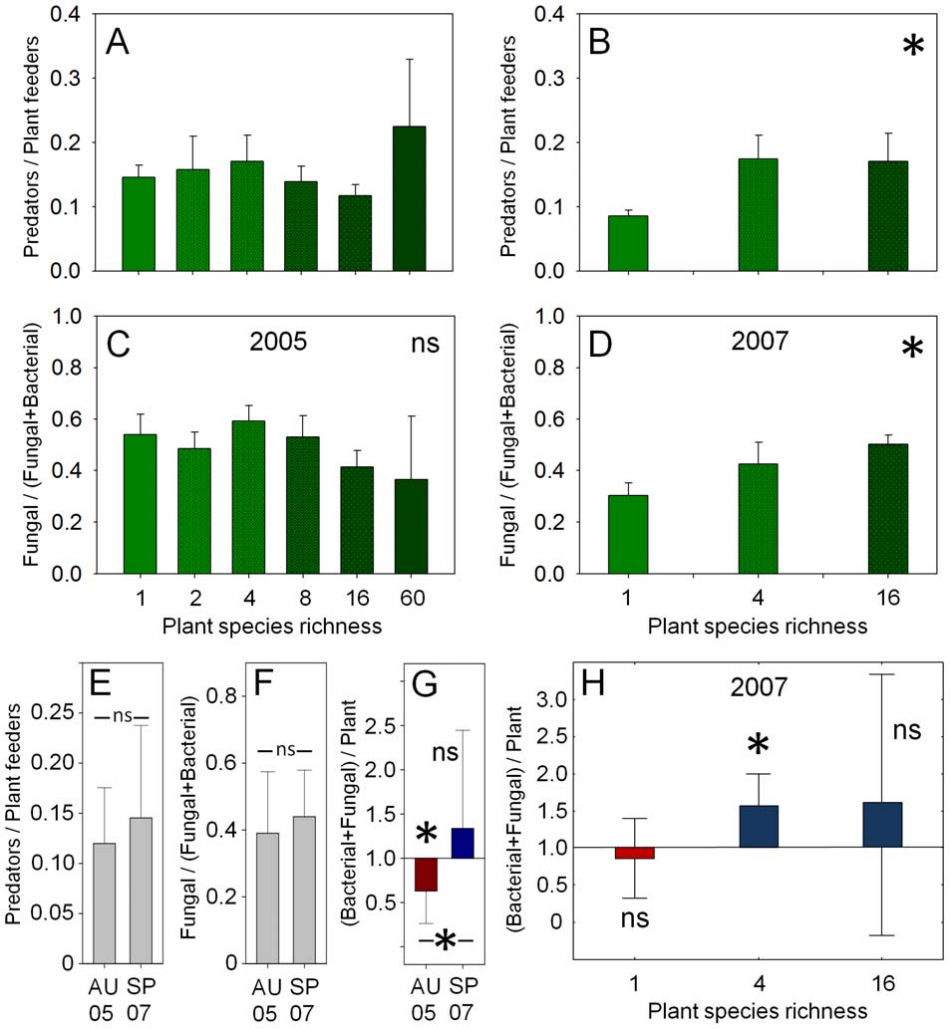
Plant diversity effects on the functional composition of nematode communities. Effect of plant species richness on the ratio between predators and plant feeders in (A) autumn 2005 and (B) spring 2007, and between fungal and bacterial feeders in (C) autumn 2005 and (D) spring 2007. Darker shading of green indicates increasing plant species richness. Ratio between (E) predators and plant feeders, (F) fungal and bacterial feeders, and (G) microbivore nematodes (bacterial and fungal feeders) and plant feeders in autumn 2005 and spring 2007. (H) Ratio between microbivore nematodes and plant feeders in spring 2007 as affected by plant species richness. The horizontal line in (G) and (H) represents a ratio of 1.0, i.e. neutral net effects of nematodes on plant productivity [38], [39]. Ratios <1.0 indicate negative net effects (lower plant productivity; given in red), and ratios >1.0 indicate positive net effects (higher plant productivity; given in blue). Levels of significance on lines (E, F, G) indicate differences between years (Wilcoxon Matched Pairs Test) and those on bars (G, H) differences from 1.0 (one-sample *t*-test). Means ± standard deviation; ns  =  not significant, *  =  significant (*P*≤0.05).

Plant community properties did not significantly affect the maturity index (2.52±0.54; mean ± standard deviation), channel index (54.60±36.76), enrichment index (56.02±19.79), structure index (66.86±20.74), ratio between predators and plant feeders, fungal and bacterial feeders, and the ratio between microbivores and plant feeders in 2005 (Table 2, Fig. 4A, C). Assigning Tylenchidae to fungal feeders did not change the results (not shown). In 2007 the ratio between predators and plant feeders, as well as that of fungal and bacterial feeders increased significantly with increasing plant species richness (Table 2, Fig. 4B, D). While the ratio between predators and plant feeders, as well as that of fungal and bacterial feeders did not differ significantly between 2005 and 2007 (*Z*_1,18_ = 0.47, *P* = 0.64 and *Z*_1,18_ = 1.16, *P* = 0.25, respectively; Fig. 4E, F), the ratio between microbivores and plant feeders increased significantly from 2005 to 2007 (*Z*_1,18_ = 2.43, *P* = 0.015; Fig. 4G). The ratio between microbivores and plant feeders was significantly lower than 1.0 in 2005 (*t*_1,18_ = −4.51, *P*<0.001), whereas it was somewhat, though not significantly, higher than 1.0 in 2007 (*t*_1,18_ = 1.36, *P* = 0.19; Fig. 4G). The elevated ratio in 2007 was primarily due to plant communities containing four species being significantly higher than 1.0 (*t*_1,4_ = 3.01, *P* = 0.030), whereas the ratio did not differ significantly from 1.0 in monocultures (*t*_1,6_ = −0.71, *P* = 0.50) and in 16 species mixtures (*t*_1,5_ = 0.88, *P* = 0.41; Fig. 4H).

## Discussion

Generally, the functional indices after three years (channel index, enrichment index and structure index) indicate a fungal dominated system with rather high values for grassland [43]; however, fungal markers were highly variable and not affected significantly by plant community properties. In contrast to our expectations (hypothesis 1), the results suggest that simplified of plant communities are associated with fewer common nematode species. Moreover and in accordance to our hypothesis (2), the results suggest that five years after establishment of the experiment the functional structure of soil food webs in simplified plant communities differs from that of species-rich plant communities, with higher ratio between predators and plant feeders and higher ratio between fungal and bacterial feeding nematodes at high plant species richness. Increased numbers of predators in diverse plant communities point to an increase in food web complexity after five years. Moreover, the ratio between microbivores and plant feeders increased significantly from autumn 2005 to spring 2007 suggesting that the nematode community, being more detrimental for plants in 2005, shifted towards being more beneficial in 2007 [38], [39]. This was primarily due to changes in nematode community composition in plant mixtures. It should be noted that samplings in 2005 (autumn) and 2007 (spring) were conducted during varying seasons, which may have also affected our results [44]. However, previous studies on the field site of the Jena Experiment showed that plant diversity effects on earthworms [45] and Collembola [46] were largely consistent in spring and autumn.

### Rare *versus* common species – which get lost?

There is increasing evidence that soil animal diversity relies on the diversity of plant communities [9]–[11], [23], [47], [48]. Similar to plants [27], [28], primarily rare species of aboveground invertebrates have been reported to get lost with declining plant diversity in grassland systems [7]. The loss of rare invertebrate consumers probably is due to the loss of habitat requirements and food resources with declining plant diversity; e.g., rare herbivore invertebrate species are likely to be food specialists relying on certain plant species which are more likely to be present in species-rich plant communities. If predominantly common or rare species are getting lost with declining plant species richness has different functional implications. Common, abundant and/or dominant species are likely to be more important for ecosystem functioning than rare species and/or species of low density (but see [49]). Following this reasoning, we expected that species-poor plant communities accommodate only few rare nematode species as compared to species-rich plant communities (hypothesis 1). In contrast to this assumption, results of the present study suggest that in particular common and abundant nematode species benefit from increasing plant diversity. This indicates that the increase in nematode taxon richness and Shannon-Wiener index with increasing plant diversity in this and previous studies [10], [22], [23] may have been due to taxa having an “intermediate” life history strategy (*cp*-3 group; some are rather *r*-strategists (colonizers), some are *K*-strategists (persisters)). Presumably, those taxa benefited from more diverse plant inputs to the soil system and increased microhabitat heterogeneity in complex plant assemblages [9], [10], [47] and this may also apply to plant feeding nematodes [10].

Although rare species have been shown to contribute significantly to ecosystem functioning [49], [50], the loss of common species is likely to be of greater functional importance and to be accompanied by distinct structural and functional changes in the soil food web. Further experiments, particular those reducing plant diversity experimentally, are necessary to support these conclusions.

### Belowground functional shifts

Recent biodiversity research focused on the consequences of community simplification for ecosystem functioning and services [3], [6], [51]. There is increasing evidence that the decline in plant species diversity is associated by marked changes in soil microbial biomass, respiration, substrate use efficiency, growth and enzyme activity [8], [16]–[18]. The few studies on the relationship between plant and soil animal diversity revealed inconsistent results leading Wardle [52] to conclude that above- and belowground diversity is weakly linked. However, in contrast to short-term studies [19], [53]-[55], recent long-term investigations suggest significant positive plant diversity effects on various soil animal taxa and trophic groups [9], [10], [23], [46]. In line with the results of the present study, Viketoft et al. [23] found the soil nematode community structure of grassland to change in the long-term; however, they assumed these changes to be not large enough to result in strong feedbacks to the plant community. In contrast to this conclusion, we take the distinct changes in the functional composition of the nematode community in response to plant species richness in the present study as indication for the existence of feedbacks to plants. Five years after establishment of the experimental plots the ratio between fungal and bacterial feeders increased significantly with plant species richness, suggesting that the fungal channel became more important in complex plant communities. Nematode community composition changed considerably with time from being detrimental to plants in autumn 2005 to being beneficial in spring 2007 [38], [39]. Again, this change was most pronounced in plant mixtures compared to monocultures. Moreover, the density of predators and the ratio between predators and plant feeders increased significantly with plant species richness indicating that the food web became more complex and predator – prey interactions more important. The increasing ratio between predators and plant feeders with increasing plant diversity corresponds to changes in the trophic structure of aboveground food webs [7]. Haddad et al. [7] ascribed the increasing density and diversity of predators to the high structural complexity of species-rich plant communities. They assumed that higher predator densities may be able to decrease herbivore infestation and thereby contribute to overyielding in primary productivity. The present paper indicates that similar mechanisms may also be true for belowground food webs. Overall, these changes indicate that the belowground food web became more consolidated and controlled by top-down forces with nutrient cycling being more slowly and characterized by competitive interactions among microorganisms due to reduced nutrient supply [21], [56].

At the field site of the Jena Experiment Eisenhauer et al. [40] found the application of nematicide to promote plant growth and they ascribed this to the suppression of plant feeding nematodes. However, they also suggested that other changes in soil food web structure, such as the decline in microbivores (particularly in bacterial feeders), may have contributed to the observed pattern. Indeed, nematicide application increased the ratio between microbivores and plant feeders from 1.29 in control subplots to 3.03 in nematicide subplots (*Z*_1,19_ = 3.81, *P*<0.001). This supports our conclusion that the observed increase in plant growth in nematicide treatments in the Jena experiment is indeed related to functional shifts in nematode communities. Further long-term studies and more specific manipulations of nematode functional groups are necessary to verify this conclusion.

Remarkably, the presence of certain plant functional groups never had a significant positive effect on nematode density or diversity. This indicates that positive effects of plant species richness on nematode diversity and the density of fungal feeders and predators were due to positive interactions between plant species or functional groups rather than due to the presence of individual plant functional groups.

### Conclusions

The present study indicates that simplifications of plant communities result in distinct belowground functional shifts. The decline in common nematode species and in density of nematode predators with decreasing plant diversity point to marked changes in soil food web structure. The relevance of the fungal energy channel increased with time, particularly in species-rich plant assemblages. Together with results from experimental manipulations of nematode communities at the study site [40] the results suggest that in the long term nematode communities increasingly stimulate nutrient cycling with potential beneficial effects on plants, in particular at high plant diversity. In contrast, high incidence of plant feeding nematodes in species-poor plant communities is likely to detrimentally affect plants [57].

## Supporting Information

Table S1 **List of experimental plots.**
(DOCX)

Table S2 **List of nematode taxa.**
(DOCX)
