# Supplementary material for: Changes in Plant Species Richness Induce Functional Shifts in Soil Nematode Communities in Experimental Grassland
Source: PLoS One. 2011 Sep 1;6(9):e24087. doi: 10.1371/journal.pone.0024087 (PMC3164708; doi:10.1371/journal.pone.0024087)
Supplement: Table S1 — List of experimental plots. (DOCX) [file pone.0024087.s001.docx]

**Table S1.** List of experimental plots (the Jena Experiment) sampled in 2005 and 2007 (given in bold) for soil nematodes. Given is the plot code (plot), block (BL), number of plant species (SR), number of plant functional groups (FR), presence of grasses (GR), small herbs (SH), tall herbs (TH), and legumes (LE), as well as the plant species that have been randomly assigned to the different plant community treatments.

| Plot | BL | SR | FR | GR | SH | TH | LE | Plant species |  |  |  |  | |  | |  | |  |  | |  |  |  |  |  |  |  |
| --- | --- | --- | --- | --- | --- | --- | --- | --- | --- | --- | --- | --- | --- | --- | --- | --- | --- | --- | --- | --- | --- | --- | --- | --- | --- | --- | --- |
| **B1A01** | **B1** | **16** | **4** | **1** | **1** | **1** | **1** | **Ant odo** | **Ave pub** | **Bro hor** | **Poa pra** | **Aju rep** | | **Pla lan** | | **Ran rep** | | **Tar off** | **Ant syl** | | **Car car** | **Ger pra** | **Tra pra** | **Lat pra** | **Lot cor** | **Tri cam** | **Vic cra** |
| B1A02 | B1 | 8 | 2 | 1 | 0 | 1 | 0 | Alo pra | Bro ere | Fes rub | Phl pra | Car pra | | Her sph | | Ran acr | | San off |  | |  |  |  |  |  |  |  |
| B1A03 | B1 | 8 | 3 | 1 | 1 | 0 | 1 | Cyn cri | Phl pra | Tri fla | Gle hed | Pri ver | | Ver cha | | Lot cor | | Med lup |  | |  |  |  |  |  |  |  |
| B1A04 | B1 | 4 | 4 | 1 | 1 | 1 | 1 | Fes pra | Pla lan | Cam pat | Ono vic |  | |  | |  | |  |  | |  |  |  |  |  |  |  |
| B1A05 | B1 | 2 | 1 | 0 | 0 | 0 | 1 | Med lup | Ono vic |  |  |  | |  | |  | |  |  | |  |  |  |  |  |  |  |
| **B1A06** | **B1** | **16** | **2** | **1** | **0** | **1** | **0** | **Alo pra** | **Ant odo** | **Ave pub** | **Bro hor** | **Hol lan** | | **Poa pra** | | **Poa tri** | | **Tri fla** | **Ach mil** | | **Ant syl** | **Cam pat** | **Cen jac** | **Ger pra** | **Her sph** | **Leu vul** | **Pim maj** |
| **B1A08** | **B1** | **1** | **1** | **0** | **0** | **0** | **1** | **Med var** |  |  |  |  | |  | |  | |  |  | |  |  |  |  |  |  |  |
| **B1A09** | **B1** | **1** | **1** | **1** | **0** | **0** | **0** | **Cyn cri** |  |  |  |  | |  | |  | |  |  | |  |  |  |  |  |  |  |
| **B1A11** | **B1** | **16** | **1** | **0** | **0** | **1** | **0** | **Ach mil** | **Ant syl** | **Cam pat** | **Car pra** | **Cir ole** | | **Cre bie** | | **Dau car** | | **Gal mol** | **Ger pra** | | **Her sph** | **Leu vul** | **Pas sat** | **Ran acr** | **Rum ace** | **San off** | **Tra pra** |
| B1A12 | B1 | 8 | 1 | 0 | 0 | 0 | 1 | Lat pra | Med lup | Med var | Ono vic | Tri cam | | Tri dub | | Tri hyb | | Tri pra |  | |  |  |  |  |  |  |  |
| **B1A13** | **B1** | **4** | **1** | **0** | **0** | **0** | **1** | **Lot cor** | **Med lup** | **Med var** | **Ono vic** |  | |  | |  | |  |  | |  |  |  |  |  |  |  |
| B1A14 | B1 | 8 | 4 | 1 | 1 | 1 | 1 | Luz cam | Tri fla | Leo his | Pla lan | Ant syl | | Dau car | | Tri cam | | Tri fra |  | |  |  |  |  |  |  |  |
| B1A16 | B1 | 2 | 2 | 1 | 1 | 0 | 0 | Poa pra | Pla lan |  |  |  | |  | |  | |  |  | |  |  |  |  |  |  |  |
| B1A17 | B1 | 2 | 2 | 1 | 0 | 1 | 0 | Alo pra | Dau car |  |  |  | |  | |  | |  |  | |  |  |  |  |  |  |  |
| **B1A18** | **B1** | **1** | **1** | **0** | **1** | **0** | **0** | **Pru vul** |  |  |  |  | |  | |  | |  |  | |  |  |  |  |  |  |  |
| **B1A19** | **B1** | **4** | **3** | **1** | **1** | **1** | **0** | **Arr ela** | **Luz cam** | **Pru vul** | **Cam pat** |  | |  | |  | |  |  | |  |  |  |  |  |  |  |
| **B1A20** | **B1** | **16** | **3** | **0** | **1** | **1** | **1** | **Aju rep** | **Bel per** | **Leo aut** | **Leo his** | **Pla lan** | | **Ver cha** | | **Ach mil** | | **Ger pra** | **Kna arv** | | **Ran acr** | **San off** | **Lot cor** | **Med var** | **Ono vic** | **Tri hyb** | **Tri rep** |
| **B1A21** | **B1** | **4** | **2** | **1** | **0** | **1** | **0** | **Fes pra** | **Luz cam** | **Ach mil** | **Cre bie** |  | |  | |  | |  |  | |  |  |  |  |  |  |  |
| B1A22 | B1 | 60 | 4 | 1 | 1 | 1 | 1 | Alo pra | Ant odo | Arr ela | Ave pub | Bro ere | | Bro hor | | Cyn cri | | Dac glo | Fes pra | | Fes rub | Hol lan | Luz cam | Phl pra | Poa pra | Poa tri | Tri fla |
|  |  |  |  |  |  |  |  | Aju rep | Bel per | Gle hed | Leo aut | Leo his | | Pla lan | | Pla med | | Pri ver | Pru vul | | Ran rep | Tar off | Ver cha | Ach mil | Ant syl | Cam pat | Car pra |
|  |  |  |  |  |  |  |  | Car car | Cen jac | Cir ole | Cre bie | Dau car | | Gal mol | | Ger pra | | Her sph | Kna arv | | Leu vul | Pas sat | Pim maj | Ran acr | Rum ace | San off | Tra pra |
|  |  |  |  |  |  |  |  | Lat pra | Lot cor | Med lup | Med var | Ono vic | | Tri cam | | Tri dub | | Tri hyb | Tri pra | | Tri rep | Vic cra | Tri fra |  |  |  |  |
| **B2A01** | **B2** | **4** | **4** | **1** | **1** | **1** | **1** | **Ant odo** | **Pru vul** | **Kna arv** | **Tri pra** |  | |  | |  | |  |  | |  |  |  |  |  |  |  |
| B2A02 | B2 | 2 | 1 | 1 | 0 | 0 | 0 | Fes rub | Tri fla |  |  |  | |  | |  | |  |  | |  |  |  |  |  |  |  |
| B2A03 | B2 | 60 | 4 | 1 | 1 | 1 | 1 | Alo pra | Ant odo | Arr ela | Ave pub | Bro ere | | Bro hor | | Cyn cri | | Dac glo | Fes pra | | Fes rub | Hol lan | Luz cam | Phl pra | Poa pra | Poa tri | Tri fla |
|  |  |  |  |  |  |  |  | Aju rep | Bel per | Gle hed | Leo aut | Leo his | | Pla lan | | Pla med | | Pri ver | Pru vul | | Ran rep | Tar off | Ver cha | Ach mil | Ant syl | Cam pat | Car pra |
|  |  |  |  |  |  |  |  | Car car | Cen jac | Cir ole | Cre bie | Dau car | | Gal mol | | Ger pra | | Her sph | Kna arv | | Leu vul | Pas sat | Pim maj | Ran acr | Rum ace | San off | Tra pra |
|  |  |  |  |  |  |  |  | Lat pra | Lot cor | Med lup | Med var | Ono vic | | Tri cam | | Tri dub | | Tri hyb | Tri pra | | Tri rep | Vic cra | Tri fra |  |  |  |  |
| **B2A04** | **B2** | **1** | **1** | **0** | **0** | **1** | **0** | **Ger pra** |  |  |  |  | |  | |  | |  |  | |  |  |  |  |  |  |  |
| **B2A05** | **B2** | **1** | **1** | **1** | **0** | **0** | **0** | **Fes pra** |  |  |  |  | |  | |  | |  |  | |  |  |  |  |  |  |  |
| **B2A06** | **B2** | **4** | **2** | **0** | **1** | **0** | **1** | **Pla lan** | **Tar off** | **Lat pra** | **Med lup** |  | |  | |  | |  |  | |  |  |  |  |  |  |  |
| B2A08 | B2 | 2 | 2 | 0 | 0 | 1 | 1 | Ran acr | Tri cam |  |  |  | |  | |  | |  |  | |  |  |  |  |  |  |  |
| **B2A09** | **B2** | **4** | **1** | **0** | **1** | **0** | **0** | **Aju rep** | **Pla lan** | **Pri ver** | **Pru vul** |  | |  | |  | |  |  | |  |  |  |  |  |  |  |
| **B2A10** | **B2** | **16** | **2** | **1** | **1** | **0** | **0** | **Alo pra** | **Ant odo** | **Arr ela** | **Bro ere** | **Fes pra** | | **Hol lan** | | **Phl pra** | | **Poa pra** | **Bel per** | | **Leo aut** | **Leo his** | **Pla lan** | **Pri ver** | **Pru vul** | **Ran rep** | **Ver cha** |
| B2A12 | B2 | 8 | 1 | 0 | 0 | 1 | 0 | Ant syl | Gal mol | Ger pra | Her sph | Kna arv | | Leu vul | | Ran acr | | San off |  | |  |  |  |  |  |  |  |
| **B2A13** | **B2** | **1** | **1** | **0** | **1** | **0** | **0** | **Pla lan** |  |  |  |  | |  | |  | |  |  | |  |  |  |  |  |  |  |
| B2A14 | B2 | 8 | 4 | 1 | 1 | 1 | 1 | Luz cam | Phl pra | Leo his | Ver cha | Kna arv | | San off | | Tri dub | | Tri hyb |  | |  |  |  |  |  |  |  |
| **B2A15** | **B2** | **1** | **1** | **0** | **0** | **0** | **1** | **Ono vic** |  |  |  |  | |  | |  | |  |  | |  |  |  |  |  |  |  |
| **B2A16** | **B2** | **4** | **3** | **0** | **1** | **1** | **1** | **Leo aut** | **Pla med** | **Kna arv** | **Vic cra** |  | |  | |  | |  |  | |  |  |  |  |  |  |  |
| **B2A18** | **B2** | **16** | **4** | **1** | **1** | **1** | **1** | **Alo pra** | **Bro hor** | **Cyn cri** | **Poa pra** | **Aju rep** | | **Pla med** | | **Pri ver** | | **Ran rep** | | **Ant syl** | **Cam pat** | **Car pra** | **Ger pra** | **Med lup** | **Tri cam** | **Tri dub** | **Tri rep** |
| B2A19 | B2 | 2 | 1 | 0 | 1 | 0 | 0 | Pla med | Tar off |  |  |  | |  | |  | |  |  | |  |  |  |  |  |  |  |
| B2A20 | B2 | 2 | 2 | 0 | 1 | 0 | 1 | Pla lan | Tri dub |  |  |  | |  | |  | |  |  | |  |  |  |  |  |  |  |
| B2A21 | B2 | 8 | 3 | 0 | 1 | 1 | 1 | Leo his | Pla med | Cre bie | Gal mol | San off | | Lot cor | | Med lup | | Ono vic |  | |  |  |  |  |  |  |  |
| **B2A22** | **B2** | **16** | **3** | **1** | **0** | **1** | **1** | **Cyn cri** | **Fes pra** | **Phl pra** | **Poa tri** | **Tri fla** | | **Ach mil** | | **Cam pat** | | **Cen jac** | **Rum ace** | | **San off** | **Lat pra** | **Lot cor** | **Ono vic** | **Tri hyb** | **Tri rep** | **Vic cra** |
| B3A01 | B3 | 1 | 1 | 0 | 0 | 1 | 0 | Gal mol |  |  |  |  | |  | |  | |  |  | |  |  |  |  |  |  |  |
| B3A02 | B3 | 2 | 2 | 1 | 0 | 1 | 0 | Fes pra | Car car |  |  |  | |  | |  | |  |  | |  |  |  |  |  |  |  |
| B3A03 | B3 | 4 | 3 | 1 | 1 | 0 | 1 | Phl pra | Pla med | Tri hyb | Vic cra |  | |  | |  | |  |  | |  |  |  |  |  |  |  |
| B3A04 | B3 | 8 | 1 | 1 | 0 | 0 | 0 | Alo pra | Arr ela | Cyn cri | Dac glo | Fes rub | | Hol lan | | Poa tri | | Tri fla |  | |  |  |  |  |  |  |  |
| B3A05 | B3 | 8 | 3 | 1 | 0 | 1 | 1 | Ant odo | Bro ere | Poa tri | Ant syl | Leu vul | | Lot cor | | Ono vic | | Tri hyb |  | |  |  |  |  |  |  |  |
| B3A06 | B3 | 1 | 1 | 1 | 0 | 0 | 0 | Fes rub |  |  |  |  | |  | |  | |  |  | |  |  |  |  |  |  |  |
| B3A07 | B3 | 8 | 4 | 1 | 1 | 1 | 1 | Bro hor | Hol lan | Pri ver | Ran rep | Her sph | | Leu vul | | Med lup | | Ono vic |  | |  |  |  |  |  |  |  |
| B3A09 | B3 | 16 | 1 | 1 | 0 | 0 | 0 | Alo pra | Ant odo | Arr ela | Ave pub | Bro ere | | Bro hor | | Cyn cri | | Dac glo | Fes pra | | Fes rub | Hol lan | Luz cam | Phl pra | Poa pra | Poa tri | Tri fla |
| B3A12 | B3 | 1 | 1 | 0 | 0 | 0 | 1 | Lat pra |  |  |  |  | |  | |  | |  |  | |  |  |  |  |  |  |  |
| B3A13 | B3 | 4 | 1 | 1 | 0 | 0 | 0 | Alo pra | Ant odo | Bro ere | Poa tri |  | |  | |  | |  |  | |  |  |  |  |  |  |  |
| B3A16 | B3 | 16 | 2 | 0 | 1 | 0 | 1 | Aju rep | Gle hed | Leo his | Pla med | Pru vul | | Ran rep | | Tar off | | Ver cha | Lat pra | | Med lup | Ono vic | Tri cam | Tri hyb | Tri rep | Vic cra | Tri fra |
| B3A17 | B3 | 1 | 1 | 0 | 1 | 0 | 0 | Ver cha |  |  |  |  | |  | |  | |  |  | |  |  |  |  |  |  |  |
| B3A19 | B3 | 2 | 2 | 1 | 1 | 0 | 0 | Tri fla | Tar off |  |  |  | |  | |  | |  |  | |  |  |  |  |  |  |  |
| B3A20 | B3 | 8 | 2 | 0 | 0 | 1 | 1 | Cam pat | Car pra | Her sph | Kna arv | Lot cor | | Tri cam | | Tri hyb | | Tri fra |  | |  |  |  |  |  |  |  |
| B3A22 | B3 | 16 | 4 | 1 | 1 | 1 | 1 | Ant odo | Bro ere | Fes rub | Phl pra | Aju rep | | Bel per | | Ran rep | | Ver cha | Cre bie | | Gal mol | Ger pra | Rum ace | Ono vic | Tri dub | Vic cra | Tri fra |
| B3A23 | B3 | 4 | 4 | 1 | 1 | 1 | 1 | Bro hor | Ran rep | Leu vul | Tri fra |  | |  | |  | |  |  | |  |  |  |  |  |  |  |
| B3A24 | B3 | 16 | 3 | 1 | 1 | 0 | 1 | Ant odo | Arr ela | Ave pub | Bro hor | Fes pra | | Poa tri | | Aju rep | | Gle hed | Pru vul | | Ran rep | Tar off | Lot cor | Med var | Tri pra | Tri rep | Vic cra |
| B4A01 | B4 | 60 | 4 | 1 | 1 | 1 | 1 | Alo pra | Ant odo | Arr ela | Ave pub | Bro ere | | Bro hor | | Cyn cri | | Dac glo | Fes pra | | Fes rub | Hol lan | Luz cam | Phl pra | Poa pra | Poa tri | Tri fla |
|  |  |  |  |  |  |  |  | Aju rep | Bel per | Gle hed | Leo aut | Leo his | | Pla lan | | Pla med | | Pri ver | Pru vul | | Ran rep | Tar off | Ver cha | Ach mil | Ant syl | Cam pat | Car pra |
|  |  |  |  |  |  |  |  | Car car | Cen jac | Cir ole | Cre bie | Dau car | | Gal mol | | Ger pra | | Her sph | Kna arv | | Leu vul | Pas sat | Pim maj | Ran acr | Rum ace | San off | Tra pra |
|  |  |  |  |  |  |  |  | Lat pra | Lot cor | Med lup | Med var | Ono vic | | Tri cam | | Tri dub | | Tri hyb | Tri pra | | Tri rep | Vic cra | Tri fra |  |  |  |  |
| B4A03 | B4 | 1 | 1 | 0 | 1 | 0 | 0 | Bel per |  |  |  |  | |  | |  | |  |  | |  |  |  |  |  |  |  |
| B4A04 | B4 | 4 | 4 | 1 | 1 | 1 | 1 | Arr ela | Pla lan | Ant syl | Tri cam |  | |  | |  | |  |  | |  |  |  |  |  |  |  |
| B4A06 | B4 | 8 | 1 | 0 | 1 | 0 | 0 | Aju rep | Bel per | Gle hed | Leo aut | Pri ver | | Pru vul | | Tar off | | Ver cha |  | |  |  |  |  |  |  |  |
| B4A07 | B4 | 4 | 2 | 0 | 0 | 1 | 1 | Car pra | Cre bie | Med lup | Tri rep |  | |  | |  | |  |  | |  |  |  |  |  |  |  |
| B4A08 | B4 | 8 | 2 | 1 | 1 | 0 | 0 | Ant odo | Ave pub | Bro hor | Fes rub | Aju rep | | Pla lan | | Tar off | | Ver cha |  | |  |  |  |  |  |  |  |
| B4A09 | B4 | 1 | 1 | 0 | 0 | 0 | 1 | Tri rep |  |  |  |  | |  | |  | |  |  | |  |  |  |  |  |  |  |
| B4A10 | B4 | 8 | 3 | 1 | 1 | 1 | 0 | Bro ere | Fes pra | Aju rep | Pla med | Pri ver | | Ach mil | | Car car | | Pim maj |  | |  |  |  |  |  |  |  |
| B4A11 | B4 | 4 | 3 | 1 | 0 | 1 | 1 | Tri fla | Her sph | Tra pra | Med var |  | |  | |  | |  |  | |  |  |  |  |  |  |  |
| B4A12 | B4 | 1 | 1 | 1 | 0 | 0 | 0 | Poa pra |  |  |  |  | |  | |  | |  |  | |  |  |  |  |  |  |  |
| B4A13 | B4 | 1 | 1 | 0 | 0 | 1 | 0 | Cir ole |  |  |  |  | |  | |  | |  |  | |  |  |  |  |  |  |  |
| B4A15 | B4 | 2 | 2 | 0 | 1 | 0 | 1 | Ran rep | Med lup |  |  |  | |  | |  | |  |  | |  |  |  |  |  |  |  |
| B4A16 | B4 | 8 | 4 | 1 | 1 | 1 | 1 | Phl pra | Poa tri | Pri ver | Tar off | Ant syl | | San off | | Tri dub | | Tri fra |  | |  |  |  |  |  |  |  |
| B4A17 | B4 | 2 | 1 | 0 | 0 | 1 | 0 | Dau car | Her sph |  |  |  | |  | |  | |  |  | |  |  |  |  |  |  |  |
| B4A18 | B4 | 16 | 4 | 1 | 1 | 1 | 1 | Alo pra | Bro hor | Cyn cri | Luz cam | Leo aut | | Pla med | | Tar off | | Ver cha | Car car | | Cre bie | Her sph | Pim maj | Lat pra | Ono vic | Tri cam | Tri hyb |
| B4A20 | B4 | 16 | 2 | 0 | 0 | 1 | 1 | Ant syl | Cam pat | Car pra | Cen jac | Cir ole | | Ger pra | | Rum ace | | Tra pra | Med var | | Tri cam | Tri dub | Tri hyb | Tri pra | Tri rep | Vic cra | Tri fra |
| B4A21 | B4 | 2 | 2 | 0 | 0 | 1 | 1 | Dau car | Med var |  |  |  | |  | |  | |  |  | |  |  |  |  |  |  |  |
| B4A22 | B4 | 4 | 1 | 0 | 0 | 1 | 0 | Cam pat | Car pra | Ger pra | Kna arv |  | |  | |  | |  |  | |  |  |  |  |  |  |  |
|  |  |  |  |  |  |  |  |  |  |  |  |  | |  | |  | |  |  | |  |  |  |  |  |  |  |
| Alo pra | *Alopecurus pratensis* (GR) | | | | | |  |  |  |  |  |  | | | | | | |  | |  |  |  |  |  |  |  |
| Ant odo | *Anthoxanthum odoratum* (GR) | | | | | | |  |  |  |  |  | | | | | | |  | |  |  |  |  |  |  |  |
| Arr ela | *Arrhenatherum elatius* (GR) | | | | | |  |  |  |  |  |  | | | | |  | |  | |  |  |  |  |  |  |  |
| Ave pub | *Avenula pubescens* (GR) | | | | | |  |  |  |  |  |  | | | | |  | |  | |  |  |  |  |  |  |  |
| Bro ere | *Bromus erectus* (GR) | | | | |  |  |  |  |  |  |  | | | | |  | |  | |  |  |  |  |  |  |  |
| Bro hor | *Bromus hordeaceus* (GR) | | | | | |  |  |  |  |  |  | | | | |  | |  | |  |  |  |  |  |  |  |
| Cyn cri | *Cynosurus cristatus* (GR) | | | | | |  |  |  |  |  |  |  | |  | |  | |  | |  |  |  |  |  |  |  |
| Dac glo | *Dactylis glomerata* (GR) | | | | | |  |  |  |  |  |  |  | |  | |  | |  | |  |  |  |  |  |  |  |
| Fes pra | *Festuca pratensis* (GR) | | | | |  |  |  |  |  |  |  |  | |  | |  | |  | |  |  |  |  |  |  |  |
| Fes rub | *Festuca rubra* (GR) | | | | |  |  |  |  |  |  |  |  | |  | |  | |  | |  |  |  |  |  |  |  |
| Hol lan | *Holcus lanatus* (GR) | | | | |  |  |  |  |  |  |  |  | |  | |  | |  | |  |  |  |  |  |  |  |
| Luz cam | *Luzula campestris* (GR) | | | | | |  |  |  |  |  |  |  | |  | |  | |  | |  |  |  |  |  |  |  |
| Phl pra | *Phleum pratense* (GR) | | | | |  |  |  |  |  |  |  |  | |  | |  | |  | |  |  |  |  |  |  |  |
| Poa pra | *Poa pratensis* (GR) | | | | |  |  |  |  |  |  |  |  | |  | |  | |  | |  |  |  |  |  |  |  |
| Poa tri | *Poa trivialis* (GR) | | | |  |  |  |  |  |  |  |  |  | |  | |  | |  | |  |  |  |  |  |  |  |
| Tri fla | *Trisetum flavescens* (GR) | | | | | |  |  |  |  |  |  |  | |  | |  | |  | |  |  |  |  |  |  |  |
| Aju rep | *Ajuga reptans* (SH) | | | | | |  |  |  |  |  |  |  | |  | |  | |  | |  |  |  |  |  |  |  |
| Bel per | *Bellis perennis* (SH) | | | | | |  |  |  |  |  |  |  | |  | |  | |  | |  |  |  |  |  |  |  |
| Gle hed | *Glechoma hederacea* (SH) | | | | | | |  |  |  |  |  |  | |  | |  | |  | |  |  |  |  |  |  |  |
| Leo aut | *Leontodon autumnalis* (SH) | | | | | | | |  |  |  |  |  | |  | |  | |  | |  |  |  |  |  |  |  |
| Leo his | *Leontodon hispidus* (SH) | | | | | | |  |  |  |  |  |  | |  | |  | |  | |  |  |  |  |  |  |  |
| Pla lan | *Plantago lanceolata* (SH) | | | | | | |  |  |  |  |  |  | |  | |  | |  | |  |  |  |  |  |  |  |
| Pla med | *Plantago media* (SH) | | | | | |  |  |  |  |  |  |  | |  | |  | |  | |  |  |  |  |  |  |  |
| Pri ver | *Primula veris* (SH) | | | | | |  |  |  |  |  |  |  | |  | |  | |  | |  |  |  |  |  |  |  |
| Pru vul | *Prunella vulgaris* (SH) | | | | | |  |  |  |  |  |  |  | |  | |  | |  | |  |  |  |  |  |  |  |
| Ran rep | *Ranunculus repens* (SH) | | | | | | |  |  |  |  |  |  | |  | |  | |  | |  |  |  |  |  |  |  |
| Tar off | *Taraxacum officinale* (SH) | | | | | | |  |  |  |  |  |  | |  | |  | |  | |  |  |  |  |  |  |  |
| Ver cha | *Veronica chamaedrys* (SH) | | | | | | |  |  |  |  |  |  | |  | |  | |  | |  |  |  |  |  |  |  |
| Ach mil | *Achillea millefolium* (TH) | | | | | | |  |  |  |  |  |  | |  | |  | |  | |  |  |  |  |  |  |  |
| Ant syl | *Anthriscus sylvestris* (TH) | | | | | | |  |  |  |  |  |  | |  | |  | |  | |  |  |  |  |  |  |  |
| Cam pat | *Campanula patula* (TH) | | | | | | |  |  |  |  |  |  | |  | |  | |  | |  |  |  |  |  |  |  |
| Car pra | *Cardamine pratensis* (TH) | | | | | | |  |  |  |  |  |  | |  | |  | |  | |  |  |  |  |  |  |  |
| Car car | *Carum carvi* (TH) | | | | |  |  |  |  |  |  |  |  | |  | |  | |  | |  |  |  |  |  |  |  |
| Cen jac | *Centaurea jacea* (TH) | | | | | |  |  |  |  |  |  |  | |  | |  | |  | |  |  |  |  |  |  |  |
| Cir ole | *Cirsium oleraceum* (TH) | | | | | | |  |  |  |  |  |  | |  | |  | |  | |  |  |  |  |  |  |  |
| Cre bie | *Crepis biennis* (TH) | | | | | |  |  |  |  |  |  |  | |  | |  | |  | |  |  |  |  |  |  |  |
| Dau car | *Daucus carota* (TH) | | | | | |  |  |  |  |  |  |  | |  | |  | |  | |  |  |  |  |  |  |  |
| Gal mol | *Galium mollugo* (TH) | | | | | |  |  |  |  |  |  |  | |  | |  | |  | |  |  |  |  |  |  |  |
| Ger pra | *Geranium pratense* (TH) | | | | | | |  |  |  |  |  |  | |  | |  | |  | |  |  |  |  |  |  |  |
| Her sph | *Heracleum sphondylium* (TH) | | | | | | | |  |  |  |  |  | |  | |  | |  | |  |  |  |  |  |  |  |
| Kna arv | *Knautia arvensis* (TH) | | | | | |  |  |  |  |  |  |  | |  | |  | |  | |  |  |  |  |  |  |  |
| Leu vul | *Leucanthemum vulgare* (TH) | | | | | | | |  |  |  |  |  | |  | |  | |  | |  |  |  |  |  |  |  |
| Pas sat | *Pastinaca sativa* (TH) | | | | | |  |  |  |  |  |  |  | |  | |  | |  | |  |  |  |  |  |  |  |
| Pim maj | *Pimpinella major* (TH) | | | | | |  |  |  |  |  |  |  | |  | |  | |  | |  |  |  |  |  |  |  |
| Ran acr | *Ranunculus acris* (TH) | | | | | |  |  |  |  |  |  |  | |  | |  | |  | |  |  |  |  |  |  |  |
| Rum ace | *Rumex acetosa* (TH) | | | | | |  |  |  |  |  |  |  | |  | |  | |  | |  |  |  |  |  |  |  |
| San off | *Sanguisorba officinalis* (TH) | | | | | | |  |  |  |  |  |  | |  | |  | |  | |  |  |  |  |  |  |  |
| Tra pra | *Tragopogon pratensis* (TH) | | | | | | |  |  |  |  |  |  | |  | |  | |  | |  |  |  |  |  |  |  |
| Lat pra | *Lathyrus pratensis* (LE) | | | | | |  |  |  |  |  |  |  | |  | |  | |  | |  |  |  |  |  |  |  |
| Lot cor | *Lotus corniculatus* (LE) | | | | | |  |  |  |  |  |  |  | |  | |  | |  | |  |  |  |  |  |  |  |
| Med lup | *Medicago lupulina* (LE) | | | | | |  |  |  |  |  |  |  | |  | |  | |  | |  |  |  |  |  |  |  |
| Med var | *Medicago x varia* (LE) | | | | | |  |  |  |  |  |  |  | |  | |  | |  | |  |  |  |  |  |  |  |
| Ono vic | *Onobrychis viciifolia* (LE) | | | | | | |  |  |  |  |  |  | |  | |  | |  | |  |  |  |  |  |  |  |
| Tri cam | *Trifolium campestre* (LE) | | | | | | |  |  |  |  |  |  | |  | |  | |  | |  |  |  |  |  |  |  |
| Tri dub | *Trifolium dubium* (LE) | | | | | |  |  |  |  |  |  |  | |  | |  | |  | |  |  |  |  |  |  |  |
| Tri fra | *Trifolium fragiferum* (LE) | | | | | | |  |  |  |  |  |  | |  | |  | |  | |  |  |  |  |  |  |  |
| Tri hyb | *Trifolium hybridum* (LE) | | | | | |  |  |  |  |  |  |  | |  | |  | |  | |  |  |  |  |  |  |  |
| Tri pra | *Trifolium pratense* (LE) | | | | | |  |  |  |  |  |  |  | |  | |  | |  | |  |  |  |  |  |  |  |
| Tri rep | *Trifolium repens* (LE) | | | | | |  |  |  |  |  |  |  | |  | |  | |  | |  |  |  |  |  |  |  |
| Vic cra | *Vicia cracca* (LE) | | | | |  |  |  |  |  |  |  |  | |  | |  | |  | |  |  |  |  |  |  |  |
|  |  |  |  |  |  |  |  |  |  |  |  |  |  | |  | |  | |  | |  |  |  |  |  |  |  |
