# Supplementary material for: Changes in Plant Species Richness Induce Functional Shifts in Soil Nematode Communities in Experimental Grassland
Source: PLoS One. 2011 Sep 1;6(9):e24087. doi: 10.1371/journal.pone.0024087 (PMC3164708; doi:10.1371/journal.pone.0024087)
Supplement: Table S2 — List of nematode taxa. (DOCX) [file pone.0024087.s002.docx]

**Table S2.** Table of nematode taxa with trophic group affiliation (bacterial feeders, plant feeders, fungal feeders, predators and omnivores), occurrence [rare (17 taxa present in 1-14 plots), intermediate (17 taxa present in 15-30 plots) and common (17 taxa present in 31-58 plots)], and colonizer-persister grouping determined in autumn 2005 (see main text for references).

|  |  |  |  |  |
| --- | --- | --- | --- | --- |
| **Taxon** | **Trophic group** | **Occurrence** | **# Plots present** | **C-P grouping** |
| *Panagrolaimus* | Bacterial feeder | Intermediate | 17 | 1 |
| *Pelodera* | Bacterial feeder | Rare | 7 | 1 |
| *Rhabditis* | Bacterial feeder | Common | 38 | 1 |
| *Acrobeles* | Bacterial feeder | Intermediate | 16 | 2 |
| *Acrobeloides* | Bacterial feeder | Common | 36 | 2 |
| *Cephalobus* | Bacterial feeder | Intermediate | 23 | 2 |
| *Chiloplacus* | Bacterial feeder | Rare | 10 | 2 |
| *Monhystera* | Bacterial feeder | Rare | 8 | 2 |
| *Plectus* | Bacterial feeder | Common | 34 | 2 |
| *Wilsonema* | Bacterial feeder | Rare | 6 | 2 |
| *Cylindrolaimus* | Bacterial feeder | Intermediate | 15 | 3 |
| *Alaimus* | Bacterial feeder | Intermediate | 26 | 4 |
| Rhabditidae juveniles | Bacterial feeder | Intermediate | 21 | 1 |
| *Aphelenchoides* | Fungal feeder | Common | 58 | 2 |
| *Aphelenchus* | Fungal feeder | Common | 48 | 2 |
| *Ditylenchus* | Fungal feeder | Common | 47 | 2 |
| *Paraphelenchus* | Fungal feeder | Intermediate | 21 | 2 |
| *Diphtherophora* | Fungal feeder | Common | 32 | 3 |
| *Diplogaster* | Omnivore | Rare | 10 | 1 |
| *Dorylaimus* | Omnivore | Common | 39 | 4 |
| *Eudorylaimus* | Omnivore | Intermediate | 16 | 4 |
| *Pungentus* | Omnivore | Common | 35 | 4 |
| *Aporcelaimellus* | Omnivore | Rare | 6 | 5 |
| *Mesodorylaimus* | Omnivore | Intermediate | 24 | 5 |
| *Prodorylaimus* | Omnivore | Intermediate | 25 | 5 |
| Dorylaimidae juveniles | Omnivore | Intermediate | 17 | 4 |
| *Aglenchus* | Plant feeder | Common | 35 | 2 |
| *Boleodorus* | Plant feeder | Rare | 5 | 2 |
| *Coslenchus* | Plant feeder | Rare | 6 | 2 |
| *Ecphyadophora* | Plant feeder | Rare | 5 | 2 |
| *Filenchus* | Plant feeder | Common | 47 | 2 |
| *Lelenchus* | Plant feeder | Rare | 10 | 2 |
| *Malenchus* | Plant feeder | Intermediate | 16 | 2 |
| *Paratylenchus* | Plant feeder | Common | 37 | 2 |
| *Psilenchus* | Plant feeder | Rare | 6 | 2 |
| *Tylenchus* | Plant feeder | Common | 52 | 2 |
| *Criconema* | Plant feeder | Common | 31 | 3 |
| *Criconemoides* | Plant feeder | Intermediate | 29 | 3 |
| *Helicotylenchus* | Plant feeder | Common | 55 | 3 |
| *Hemicycliophora* | Plant feeder | Rare | 9 | 3 |
| *Pratylenchus* | Plant feeder | Common | 47 | 3 |
| *Rotylenchus* | Plant feeder | Common | 58 | 3 |
| *Tylenchorhynchus* | Plant feeder | Intermediate | 30 | 3 |
| *Trichodorus* | Plant feeder | Rare | 7 | 4 |
| Tylenchidae genus 1 | Plant feeder | Intermediate | 28 | 2 |
| Tylenchidae genus 2 | Plant feeder | Rare | 5 | 2 |
| *Seinura* | Predator | Intermediate | 18 | 2 |
| *Anatonchus* | Predator | Rare | 9 | 4 |
| *Mononchus* | Predator | Intermediate | 21 | 4 |
| *Mylonchulus* | Predator | Rare | 13 | 4 |
| *Discolaimus* | Predator | Rare | 5 | 5 |
|  |  |  |  |  |
